# Revealing potential molecular targets bridging colitis and colorectal cancer based on multidimensional integration strategy

**DOI:** 10.18632/oncotarget.6067

**Published:** 2015-10-10

**Authors:** Xu Guan, Ying Yi, Yan Huang, Yongfei Hu, Xiaobo Li, Xishan Wang, Huihui Fan, Guiyu Wang, Dong Wang

**Affiliations:** ^1^ Department of Colorectal Cancer Surgery, the Second Affiliated Hospital of Harbin Medical University, Harbin, China; ^2^ College of Bioinformatics Science and Technology, Harbin Medical University, Harbin, China; ^3^ Department of Pathology, Harbin Medical University, Harbin, China; ^4^ Department of Biochemistry and Molecular Biology, Shantou University Medical College, Shantou, China

**Keywords:** colorectal cancer, colitis, crosstalk, pivot, network analysis

## Abstract

Chronic inflammation may play a vital role in the pathogenesis of inflammation-associated tumors. However, the underlying mechanisms bridging ulcerative colitis (UC) and colorectal cancer (CRC) remain unclear. Here, we integrated multidimensional interaction resources, including gene expression profiling, protein-protein interactions (PPIs), transcriptional and post-transcriptional regulation data, and virus-host interactions, to tentatively explore potential molecular targets that functionally link UC and CRC at a systematic level. In this work, by deciphering the overlapping genes, crosstalking genes and pivotal regulators of both UC- and CRC-associated functional module pairs, we revealed a variety of genes (including FOS and DUSP1, etc.), transcription factors (including SMAD3 and ETS1, etc.) and miRNAs (including miR-155 and miR-196b, etc.) that may have the potential to complete the connections between UC and CRC. Interestingly, further analyses of the virus-host interaction network demonstrated that several virus proteins (including EBNA-LP of EBV and protein E7 of HPV) frequently inter-connected to UC- and CRC-associated module pairs with their validated targets significantly enriched in both modules of the host. Together, our results suggested that multidimensional integration strategy provides a novel approach to discover potential molecular targets that bridge the connections between UC and CRC, which could also be extensively applied to studies on other inflammation-related cancers.

## INTRODUCTION

Ulcerative colitis (UC) is a chronic inflammatory bowel disease associated with an increased risk of developing colorectal cancer (CRC) [1]. The cumulative risk of ulcerative colitis-associated CRC has increased by 18-20% in the 30 years since the disease was identified [2, 3]. Accumulating evidence has indicated that the process bridging UC to CRC is complex and long-term involving multiple biological mechanisms, such as inhibition of apoptosis, stimulation of angiogenesis, epithelial-mesenchymal transition and cell proliferation [4-6]. Although close connections between UC and CRC have been generally accepted, the underlying detailed mechanisms and crucial molecular targets for comprehensive understanding still require further exploration.

Over decades of intensive research, major progress has mainly been related to several key molecules such as p53, K-ras, APC, Bcl-2, NFκB, and COX-2 [7]. However, the pathogenesis underlying UC and CRC should involve a combined effect that acts through multifactorial biological process, such as gene expression alteration, transcriptional or post-transcriptional dysregulation, and even microbial intervention [8-10]. Therefore, the comprehensive exploration of the mechanism associated with this close connection should be analyzed at a whole system level rather than at the level of single isolated components. The necessity to obtain an overall and accurate understanding of the link is of significance.

For the purpose of exploring the complexity of pathogenesis in UC-CRC link, global and integrated network-based approaches should be encouraged to increase the probability of identifying potential molecular targets. To better address this issue, we introduced a multidimensional integration strategy based on gene expression profiling, protein-protein interactions (PPIs), and transcriptional and post-transcriptional regulation data to identify biologically meaningful gene modules involved in the complex connections. By deciphering the significant crosstalk between these UC- and CRC- modules, potential molecular targets were further revealed. In addition, by extracting currently curated virus-host interactions based on several data sources, we tentatively examined potential molecules in viruses. Collectively, this study provides novel insights into molecular targets involved in the pathogenesis of UC-CRC link.

## RESULTS

### Identification of functional UC- and CRC-associated modules

Four expression data sets of UC and CRC were downloaded and re-normalized using the least-variant set (LVS) algorithm [11]. With the R package siggenes [12], we computed the UC-DEGs by comparing the UC and normal samples per dataset at a FDR cutoff of 0.05. Then we kept those UC-DEGs that occurred in both datasets. The same procedure was also applied to the identification of CRC-DEGs. In total, we obtained 4474 UC- and 2545 CRC-DEGs, which were used to represent the aberrant expression underlying UC and CRC. Among them, we observed that about 30% of disease genes curated in database DisGeNET recurred as differentially expressed [13], with an even higher portion of 35% UC disease genes recurred as UC-DEGs in our data.

To comprehensively characterize the molecular mechanisms bridging UC to CRC, we mapped DEGs onto the human PPI network and then identified tightly clustered functional UC- and CRC-associated modules. In total, we acquired 79 UC modules (Supplementary Table 1) and 54 CRC modules (Supplementary Table 2). Via examining those modules with functional enrichment analysis of GO terms and KEGG pathways (Supplementary Table 3), we demonstrated that UC-associated modules tended to function in inflammation-related functions, like “innate immune response in mucosa” and “cellular response to interleukin-4”. Similarly, CRC-associated modules were important in cancer-related pathways, for example “TGF-beta signaling pathway” and “Wnt signaling pathway”. However, we also observed that certain UC-associated modules were closely correlated with cancer-related functions, like “regulation of cell proliferation” and “pathways in cancer”, and vice versa. Hence, we reasoned that our integrated approach from a systematic functional perspective might provide insights into the underlying molecular mechanisms bridging inflammation and cancer in colon.

### Dissection of the crosstalk between UC and CRC with overlapping genes

According to the functional modules we identified, 33 module pairs of UC and CRC were observed in total that significantly shared overlapping DEGs with a P value cutoff of 0.05 computed by MCODE algorithm (Supplementary Table 4). Overlapping genes have been extensively recognized as versatile factors involved in a variety of phenotypically diverse disease states [14, 15]. We therefore explored those overlapping DEGs to produce the first dimension explanation of the connections.

**Figure 1:**
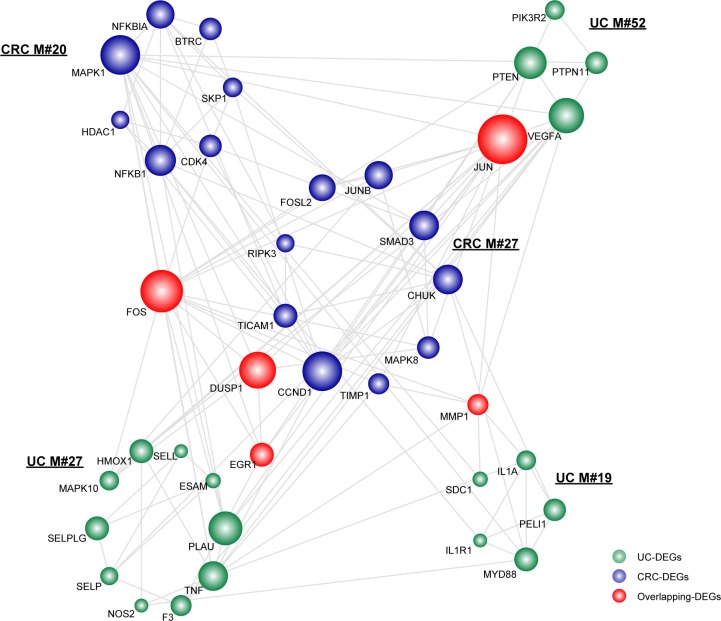
The significant overlapping module subnetwork Each module was extracted after mapping DEGs to the human PPI network using the MCODE program. The overlapping significance of module pairs of inflammation and cancer was determined by a hypergeometric test with a cutoff of 0.05. Network nodes are colored to show that they are inflammation, cancer or overlapping DEGs. Node size is shown according to its network degree. Module number is marked besides the module.

As indicated, we observed three UC-associated modules and two CRC-associated modules sharing overlapping DEGs, which contained the genes FOS, JUN, DUSP1, EGR1 and MMP1 (Figure 1). Among those overlapping DEGs, proteins encoded by JUN and FOS are major components of the transcription factor complex activated protein 1 (AP-1), which are involved in balancing immune responses. As such, they significantly contributed to inflammatory stimuli [16]. Additionally, as previously reported, their abnormal activation via oncogenic signaling pathways is closely associated with enhanced cell proliferation, cell mobility, invasiveness and therefore malignant transformation in CRC [17, 18]. According to our results, the genes JUN and FOS with a relatively larger degree in the module subnetwork (Figure 1), thus functionally linked UC and CRC.

Other overlapping genes, such as matrix metalloproteinase 1 (MMP1), a member of the protease family, functions in the degradation of components of the extracellular matrix (ECM) [19]. MMPs contribute to inflammation through matrix remodeling, in pathways involving components such as chemokines, growth factors and proteases. Specifically, in CRC, the aberrant activation of MMP-1 correlates with advanced stage, lymph node metastasis and poor prognosis [20]. As for the remaining two overlapping DEGs (i.e., EGR1 and DUSP1), they have not been reported to be involved in the tight connections. EGR1 is a tumor suppressor and is regulated by many different stimuli such as cytokines, growth factors, and apoptosis-promoting factors [21]. Accordingly, the gene has been reported to be involved in diverse biological processes, such as cell growth, cell differentiation, wound healing and apoptosis. The other gene, DUSP1, functions as a pro-inflammatory mediator and has been found in head and neck squamous cell carcinoma, which could in turn be stimulated by multiple pro- and anti-inflammatory stimuli [22, 23]. Accordingly, further experiments on the precise functions of EGR1 and DUSP1 in liking UC and CRC are still needed, which would definitely help to understand the underlying molecular mechanisms.

### Crosstalk interactions bridging the link between UC and CRC

In addition to those significantly overlapping UC- and CRC-associated modules, we also observed 46 module pairs with significant crosstalk interactions in comparison with a random distribution (Supplementary Table 5). These crosstalk interactions might bridge UC and CRC through participating in correlated GO categories or KEGG pathways, which thus brings us to the second dimension of interpretation of the link.

**Figure 2:**
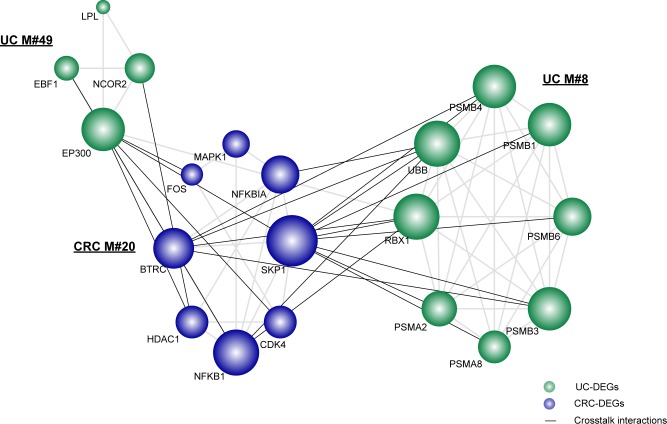
The significant crosstalk module subnetwork The significant crosstalk module pairs were computed in comparison with 1000 random networks, with a p value cutoff of 0.05. Nodes are colored as inflammation or cancer DEGs. Crosstalk interactions are shown in black. Node size is shown according to its network degree.

As shown, there were two UC-associated modules and one CRC-associated module tightly connected with each other via significant crosstalk interactions (Figure 2). Accordingly, we observed that the gene EP300 from the UC-associated module and the gene SKP1 from the CRC-associated module were highly responsible for the significant crosstalk among the three functional modules. KEGG analysis indicated that SKP1 and EP300 were involved in the Wnt signaling pathway and TGF-beta signaling pathway, which are tightly correlated with the establishment of a local inflammatory micro-environment and the progression of CRC [24]. In combination with GO analysis, we demonstrated that SKP1 and EP300 were also correlated with the regulation of the cell cycle. It is therefore plausible that the significant crosstalk between UC- and CRC-associated modules might employ the above biological functions to form the local link bridging UC and CRC. We showed that the gene BTRC, another component of the Wnt signaling pathway, had a relatively large degree of crosstalk interactions and might be a potential mediator underlying the connections. Moreover, as reported previously, SKP1 connects cell cycle regulators to proteolysis machinery between the UC module (M#8) and the CRC module (M#20) in our significant crosstalk subnetwork [25]. Collectively, those module genes, despite not being shared as overlapping genes between UC and CRC, serve as different components of the same or similar functional categories or pathways, which help to bridge UC and CRC via significant crosstalk interactions.

### Pivot regulators correlate with module subnetworks linking UC and CRC

Transcriptional and post-transcriptional regulations have long been involved in the initiation and progression of UC and CRC. However, the intricate regulatory mechanisms underlying the connections have not been clearly explored, which leads to the third dimension describing the underlying link. Combining those identified functional modules, TF-target and miRNA-target interactions, we identified significant regulators of both UC- and CRC-associated modules, termed as pivot regulators (Supplementary Table 6). In total, we identified 105 pivot TFs and 255 pivot miRNAs. In comparison with disease miRNAs curated in database miR2Disease [26], We found that, approximately 54% of these validated UC- and/or CRC-related miRNAs were identified as pivot miRNAs in our data, which therefore implied that those pivot regulators might be potentially important in bridging UC and CRC.

**Figure 3:**
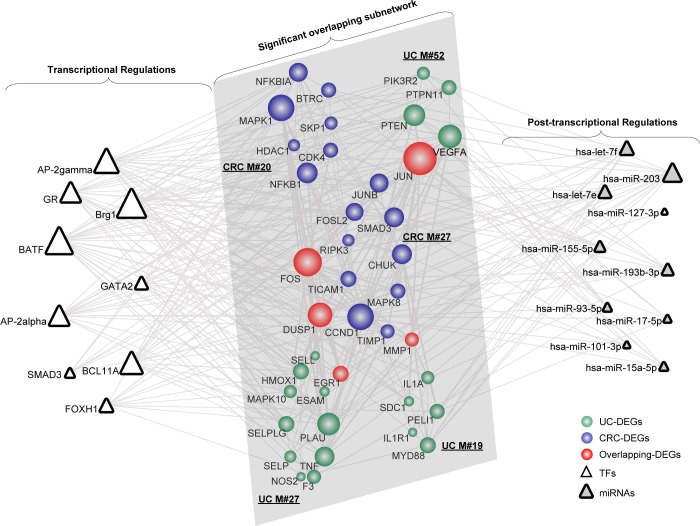
The significant overlapping module subnetwork manipulated by pivot regulators Pivot regulators, both TFs and miRNAs, were computed based on the number of their interactions with the module pair and the enrichment significance of their regulating targets. Network nodes are colored as inflammation, cancer or overlapping DEGs with size showing its network degree. Pivot TFs are shown as triangles, while pivot miRNAs are triangles filled with grey.

According to the module subnetworks discussed above, we extracted pivot regulators and the corresponding regulations for the significant overlapping (Figure 1) and crosstalk subnetwork (Figure 2), respectively. As for the significant overlapping module subnetwork, we identified 9 pivot TFs and 10 pivot miRNAs, which significantly correlated with the subnetwork (Figure 3). Based on their degree distribution, we demonstrated that most pivot regulators of both TFs and miRNAs, tended to connect to those overlapping genes with a relatively greater influence on the subnetwork, as expected. Retracing originally applied miRNA-target databases (for example miRanda), we showed that those pivot miRNA regulations tended to be predicted with high-confidence, some even identified as experimentally validated (Supplementary Table 7). The overlapping gene JUN possessed the most interactions with pivot regulators, and has been shown to be a mediator between UC and CRC [27]. To uncover regulations correlating with the significant overlapping module subnetwork, we further explored corresponding pivot regulators. The transcriptional regulator SMAD3 is both a tumor suppressor and a vital mediator of TGF-β-mediated immune suppression, which likely contributes to the process bridging UC to CRC through targeting growth-related proteins [28]. The post-transcriptional pivot regulator miR-155, an inducible regulator, is tightly associated with increased cytokine production [29]. Additionally, enhanced mutation activity and aberrant cell cycle regulation have been observed based on the overexpression of miR-155 [30]. Alternatively, miR-155 might function by regulating those vital overlapping genes linking UC and CRC. Additionally, we showed that the overlapping gene DUSP1 possessed the same number of interactions with pivot regulators as the gene JUN. Therefore, DUSP1 as a potential key target, together with its interacting pivot regulators, might play vital roles underlying the link.

**Figure 4:**
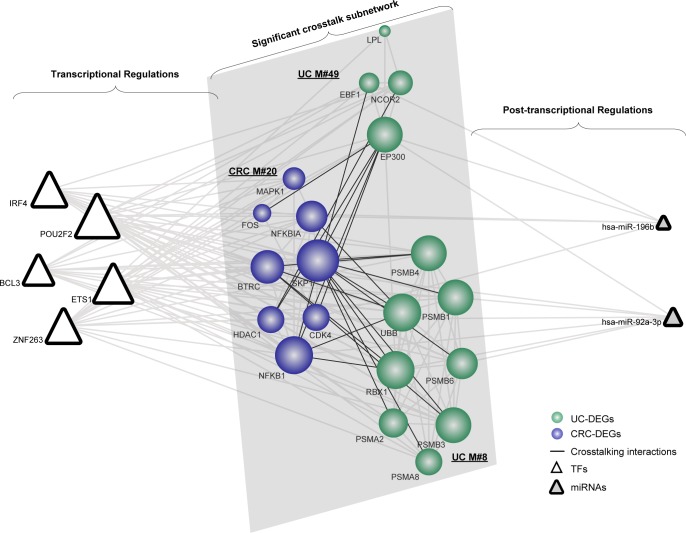
The significant crosstalk module subnetwork manipulated by pivot regulators Network nodes are colored as inflammation or cancer DEGs with size showing their network degree. Pivot TFs are shown as triangles, while pivot miRNAs are triangles filled with grey.

Similarly, based on the significant crosstalk module subnetwork, we extracted 5 pivot TFs and 2 pivot miRNAs (Figure 4). In our results, the gene SKP1 and the gene EP300 are responsible for the functional crosstalk between the three modules. Thus, we further explored their related pivot regulators correlating with the module subnetwork. As indicated, SKP1 tended to be regulated by pivot TFs, whereas EP300 was regulated by both miRNAs and TFs. The transcriptional regulator ETS1 has been suggested to function in the induction of cellular differentiation and the regulation of cell growth in CRC [31]. Additionally, an emerging role for genes involved in immune cell function has been observed, which is crucial in the regulation of inflammatory and anti-inflammatory responses [32]. The post-transcriptional regulator miR-196b has been previously shown to be associated with aggressive progression and poor clinical outcomes in CRC [33]. Moreover, its key role in the onset or relapse of UC has also been observed, which therefore supports its functions underlying the link [34]. Collectively, we concluded that the validated or novel pivot regulators are important in manipulating these significant overlapping or crosstalk module subnetworks.

### Virus-host interactions involved in the underlying link between UC and CRC

Even though a close connection between microbial infection (bacteria and viruses) and CRC has not been fully established, current evidence supports the involvement of these organisms in oncogenesis [35]. Epstein-Barr virus (EBV) has been observed to exist extensively in UC and different stages of CRC, but the corresponding molecular mechanisms remain unexplored [36, 37]. We downloaded virus-host interactions to examine whether a virus could affect the link via binding with proteins or RNAs from host cells, in addition to the above three-dimensional molecular links between UC and CRC. According to the pivot identification method, we identified 8 viral proteins and RNAs, in 3 virus species (Supplementary Table 8).

**Figure 5:**
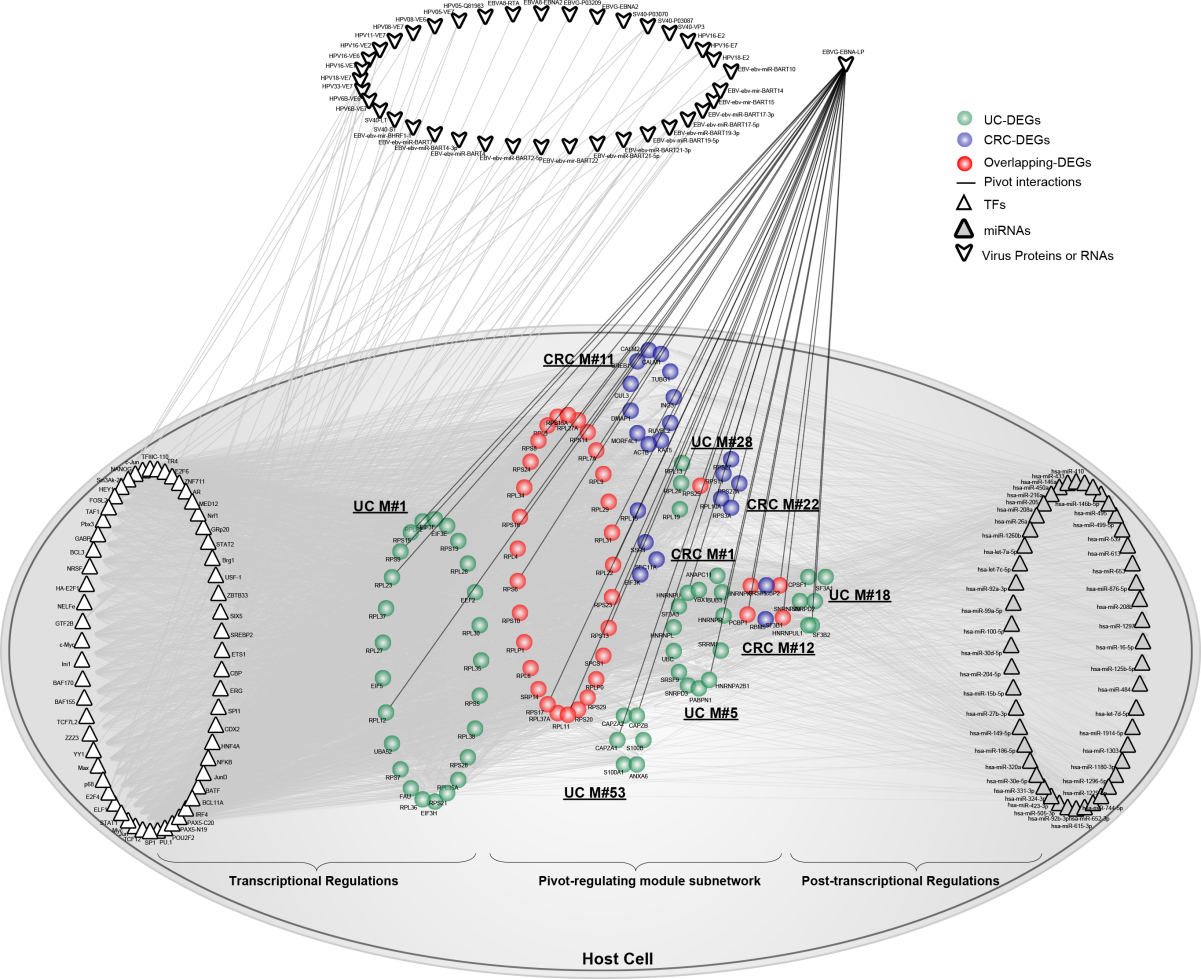
The involvement of protein EBNA-LP from EBV underlying the link between UC and CRC Based on pivot analysis, we computed pivot proteins or RNAs from viruses. As shown, pivot protein EBNA-LP from EBV could significantly regulate 9 functional modules. Together, pivot TFs and miRNAs were extracted. Other virus proteins or RNAs not serving as pivots were also shown with grey interactions with pivot TFs in the module subnetwork. Network nodes are colored as inflammation, cancer or overlapping DEGs. Pivot TFs are shown as triangles, while pivot miRNAs are triangles filled with grey. Virus proteins or RNAs are shown outside of the host cell. Pivot virus proteins interacting with the host cell are shown using black lines.

Interestingly, we observed that nuclear antigen leader protein (EBNA-LP) of EBV significantly correlated with 4 CRC-related and 5 UC-related functional modules containing 22 cancer DEGs, 53 inflammation DEGs and 34 overlapping DEGs (Figure 5). Accordingly, we extracted their related pivot TFs and miRNAs, which included 59 pivot TFs and 48 miRNAs. We also included other related virus-host interactions, which involved 15 virus miRNAs and 22 virus proteins.

Pivot EBNA-LP connected to 35 host proteins in the module subnetwork. According to GO analysis, we showed that these genes participated in the functional categories of cell death, apoptosis and the regulation of gene expression, which were tightly associated with the progression underlying both UC and CRC. In particular, host cell death triggered by various pathological stimuli turned out to be regulated by both host and pathogen, as is the case in viral infection [38]. We reasoned that cell death might be a vital function that tightly connected UC and CRC modules, but was also targeted by both the host and virus. Moreover, we also extracted other virus-host interactions associated with the module subnetwork. Unexpectedly, we found that they all targeted pivot TFs, which could thus maximally altered gene expression. Collectively, virus proteins or RNAs might be involved in the link between UC and CRC, together with pivot TFs and miRNAs, through the regulation of host cell death.

**Figure 6:**
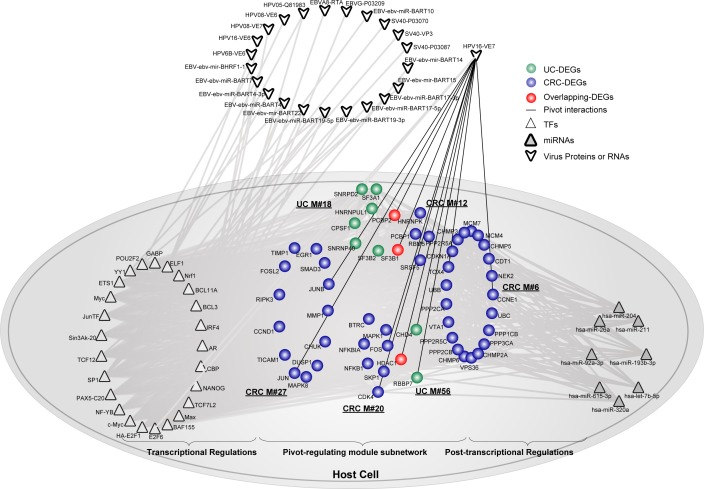
The involvement of protein E7 from HPV underlies the link between UC and CRC Pivot protein E7 from HPV significantly regulated 6 functional modules. Together, pivot TFs and miRNAs were extracted. Other virus proteins or RNAs not serving as pivots were also shown with grey interactions with pivot TFs in the module subnetwork. Network nodes are colored as inflammation, cancer or overlapping DEGs. Pivot TFs are shown as triangles, while pivot miRNAs are shown as triangles filled with grey. Virus proteins or RNAs are shown outside of the host cell. Pivot virus proteins interacting with the host cell are shown using black lines.

Additionally, we also found that human papillomavirus (HPV), which has been reported to cause more than 90% of invasive cervical cancers worldwide [39], was also identified as a significant component between the virus-host interaction in colon. We observed that protein E7 of HPV significantly connected to two UC modules and four CRC modules (Figure 6). Subsequently, 25 corresponding pivot TFs and 8 pivot miRNAs were extracted. Moreover, 10 virus proteins relating to the module subnetwork were also extracted, although they did not serve as pivots. Protein E7 is a viral oncogene for HPV and has been shown to play vital roles in diverse types of human cancers [40, 41]. The protein is known to interact with the tumor suppressor gene pRb and also its family members, which binds to TFs such as E2F and thus represses the transcription of cell-cycle related genes [42]. As validated previously, E7 was already detected in CRC samples [43, 44]. Moreover, an establishment of local immune suppression has also been observed in the epithelium of HPV-associated precancerous lesions and malignancies. Collectively, we reasoned that, together with pivot TFs and miRNAs, E7 might also play a role in linking virus and host, to form a tightly connected molecular community bridging inflammation and cancer in colon.

## DISCUSSION

In recent years, more attention has been focused on inflammatory bowel disease, especially UC, because of its role in predisposing patients to CRC. It is now widely accepted that the close connections between UC and CRC involves complex biological processes, but most of them remain unclear. For a better understanding, we should explore the potential mechanisms together with pivotal targets from a global view, instead of studying single isolated components. Hence, we established a multidimensional integration methodology based on the combination of gene expression data, PPIs, transcriptional and post-transcriptional regulation data, and host-virus interactions.

To systematically reveal potential molecular targets that could be responsible for bridging UC and CRC, we firstly examined the overlapping genes between UC- and CRC-associated modules, which usually play essential roles in many biological processes. As demonstrated, we identified five overlapping genes; some of them, such as JUN, FOS and MMP1, have been experimentally verified as functionally significant underlying the connections [16-19]. Secondly, we examined significant crosstalk module pairs. Although crosstalk genes, for example EP300 and SKP1, existed in separate UC- and CRC-associated modules, they were functionally connected by crosstalk interactions and involved in the same representative pathways: the Wnt signaling pathway or the TGF-beta signaling pathway. Thirdly, TFs and miRNAs usually control genes in a cooperative manner to guarantee the accuracy of gene expression. Hence, TFs and miRNAs with their targets could form an integrated regulation network. Based on the significant overlapping and crosstalk module subnetworks, we explored the functions of those tightly correlated pivot regulators underlying the connections. We showed that TF SMAD3 and miR-155 simultaneously regulated the overlapping gene JUN, and have been validated for their importance in the tight links between UC and CRC [28-30]. Finally, accumulating evidence has been observed that viral DNA or antibodies were presented in patients with UC and CRC [37, 39, 40, 43]. However, those viral molecule involved in the regulation of this process in the host have never been reported. Hence, we attempted to explore the viral proteins and miRNAs that significantly crosstalked with UC- and CRC-associated module pairs in the host. Collectively, we suggested that those separate levels might be employed by UC to functionally connect to CRC, individually or together, which therefore help to comprehensively deepen our understanding of the complex connections between UC and CRC.

In order to obtain UC- and CRC-associated functional modules, we identified UC- and CRC-DEGs, separately. As being limited by currently curated expression profiles, we are lack of datasets transforming from UC to CRC, which means they examined gene expression of UC patients and then re-examined the same patients developing CRC later. Thus, in order to decipher the underlying functional connections, we made a detour to identify DEGs separately, instead of computing DEGs directly between inflammation and cancer or overlapped between UC- and CRC-DEGs, even though we recommend it as a good complement to our integrated approach, which might help gain more insights into the underlying connections.

In conclusion, our work provides novel insights into a multidimensional integration network that explores potential molecular targets involved in the links between UC and CRC. With the increasing diversity of high throughput data, this multidimensional integration network is expected to have more confidence and effectiveness in elucidating the underlying biological mechanisms, not only for studies on the tight connections between UC and CRC, but also for other inflammation-associated cancers, such as hepatic carcinoma [45], gastric carcinoma [46] and esophageal carcinoma [47]. Furthermore, with the exception of those experimentally validated molecules recurred, more efforts should be made to study the remaining potential molecular targets (for example, gene DUSP1 and EP300; pivot miRNA miR-193b and miR-15a; pivot TF BCL11A and BCL3; pivot viral protein EBNA-LP and E7), which may also contribute to the discovery of more detailed molecular mechanisms and provide theoretical guidance for biological research in the future.

## MATERIALS AND METHODS

### Data resources

We collected four expression microarray datasets from the NCBI Gene Expression Omnibus (GEO), which included GSE20916 [48], GSE23878 [49], GSE38713 [50] and GSE47908 [51] (Table 1), according to rules 1) examined using the same platform to reduce inter-dataset variances, 2) contain at least two conditions, while normal status is essential per dataset. These four datasets were generated on the same platform of Affymetrix Human Genome U133 Plus 2.0 Array which included 54675 probe sets and 38500 genes.

**Table 1:** The microarray datasets analyzed in this study

Accession ID	Samples			Platform	Submission date	References
	Normal	UC	CRC			
GSE20916	24	-	36	HG-U133 Plus 2.0	03.16.2010	Ref 48
GSE23878	24	-	35	HG-U133 Plus 2.0	08.30.2010	Ref 49
GSE38713	13	30	-	HG-U133 Plus 2.0	06.14.2012	Ref 50
GSE47908	15	45	-	HG-U133 Plus 2.0	06.13.2013	Ref 51

Human PPIs were deposited in the STRING (Search Tool for the Retrieval of Interacting Genes/Proteins, http://string-db.org/) database (Release 9.1), which helped us to comprehensively uncover and annotate functional interactions in living systems [52]. Based on the score cutoff of 0.90, we retrieved 9061 proteins with 69400 interactions in humans.

As for the regulatory relationship, we downloaded human regulations of transcription factors (TFs) from ChIPBase (http://deepbase.sysu.edu.cn/chipbase/), which formed a dense regulatory network containing 120 unique TFs together with 324007 regulatory interactions [53]. In consideration of other key regulators, we downloaded combined miRNA-target interaction data from the database miRTarBase (http://mirtarbase.mbc.nctu.edu.tw/), the RNA-associated interaction database (RAID) (http://www.rna-society.org/raid/), and the miRanda (http://www.microrna.org/microrna/home.do) database [54-56]. In total, we included 733 miRNAs and 376385 interactions with 24195 experimentally verified and 352190 computationally predictions for further analysis.

Virus-host interactions were collected from the three databases of ViRBase (http://www.rna-society.org/ViRBase/), VirusHostNet (http://virhostnet.prabi.fr/) and VirusMentha (http://virusmentha.uniroma2.it/) [57-59]. In combination, we generated 5662 interactions between 24 viruses and humans involving 146 proteins and 48 miRNAs of virus, as well as 2364 proteins and 21 miRNAs of humans.

In order to measure our predictions of potential candidate genes and pivot miRNAs, disease genes and miRNAs were also collected from databases. 85 UC- and/or CRC- disease genes were curated in database DisGeNET (http://www.disgenet.org/web/DisGeNET/menu/home; data source: curated data, which corresponds to associations from CTD (human data), Uniprot, and ClinVar). Besides, we obtained 95 disease miRNAs from database miR2Disease (http://www.miR2Disease.org/; disease category: UC and/or CRC).

### Identifying UC- and CRC-related functional modules

As per dataset, all CEL files were downloaded and normalized based on the least-variant set (LVS) of genes algorithm [60-63], which is robust against violation of the standard assumptions and outperforms most of other normalization approaches. To reduce the inter-dataset variances [64], we applied the same normalization algorithm across different datasets. Based on the normalized expression profile of UC and CRC, we used the R package siggenes to compute differentially expressed genes (DEGs) [12]. As for UC-DEGs, we compared UC and normal samples to identify DEGs in both datasets (i.e. GSE38713 and GSE47908)) at a FDR cutoff of 0.05, separately. And then we kept those DEGs identified as differentially expressed in both datasets. The same procedure was subsequently applied to the identification of CRC-DEGs.

After mapping those UC- and CRC-associated DEGs onto a PPI network, we could retrieve a respective UC- and CRC-associated subnetwork. With the help of the MCODE algorithm, functional clustered DEGs termed as UC- and CRC-associated modules were identified [65].

### Computing significant overlapping pairs of UC and CRC modules

$$\text{p}=1-\sum_{i=0}^{m-1}\frac{\left(\begin{array}{l} n\\ i \end{array}\right)\left(\begin{array}{l} N-n\\ M-i \end{array}\right)}{\left(\begin{array}{l} N\\ M \end{array}\right)}$$

As for the per UC- and CRC-associated module pair, we computed the significance of their overlapping DEGs using a hypergeometric test as follows:

N is the number of genes in the STRING database. M and n represent the number of genes in the CRC and UC modules, respectively, and m means the number of overlapping DEGs. The overlapping module pair was considered as significant, with a p value less than 0.05.

### Determining significant crosstalk pairs of UC and CRC modules

Significant crosstalk between UC and CRC module pairs was defined as the number of their interactions that was significantly more than that observed from a random distribution. Based on this definition, we randomized the original PPI network 1000 times by keeping the degree distribution of nodes unchanged [66]. Subsequently, random module pairs of the same size were extracted from the per random PPI network. In comparison with the random distribution, we could determine the significance for each UC and CRC module pair by counting the times when random observations were more than the real number of interactions between the module pair. All crosstalking module pairs were considered as significant, with a p value less than 0.05.

### Exploring pivot regulators

For the per UC and CRC module pair, we determined their pivot regulators as: (i) at least two regulations between the regulator and each module of the pair and (ii) significant enrichment of targets for each regulator per module with a p value cutoff of 0.05 (hypergeometric test) [67]. Moreover, we also included viral proteins and miRNAs into the analysis of pivots, according to the same rules.

## SUPPLEMENTARY TABLES
















